# Antimicrobial treatment of patients with a periprosthetic joint infection: basic principles

**DOI:** 10.1186/s42836-023-00169-4

**Published:** 2023-03-02

**Authors:** Wouter Rottier, Jessica Seidelman, Marjan Wouthuyzen-Bakker

**Affiliations:** 1 Department of Medical Microbiology and Infection Prevention, University Medical Center Groningen, University of Groningen, Groningen, 9713 GZ the Netherlands; 2 Division of Infectious Diseases, Duke University School of Medicine, Durham, NC 27708 USA

**Keywords:** Periprosthetic joint infection, Antibiotic treatment, Biofilm, Antibiotic tolerance, Biofilm active antibiotics, Oral treatment, Treatment duration

## Abstract

The antibiotic treatment of periprosthetic joint infections (PJI) is complicated by the presence of biofilm produced by bacteria on the abiotic surface of the implant. Bacteria within the deeper layers of the biofilm become metabolically less active, resulting in antibiotic tolerance due to several mechanisms. This review describes the basic principles of antibiotic treatment in PJI in relation to the behavior of bacteria within the biofilm. The concept of biofilm-active antibiotics will be explained from an *in vitro* as well as *in vivo* perspective. Evidence from clinical studies on biofilm-active antibiotics in PJI will be highlighted, mainly focusing on the role of rifampicin for Gram-positive microorganisms and fluoroquinolones for Gram-negative microorganisms. The optimal treatment duration will be discussed as the timing of switching to oral antibiotic therapy.

## Introduction

Periprosthetic joint infection (PJI) is characterized by the formation of biofilm on the implanted foreign body [1]. Biofilm consists of bacteria (in some cases yeasts) surrounded by an extracellular matrix and offers a protective environment for the microorganisms present [2]. These microorganisms enter a dormant state and, consequently, antimicrobial agents cannot exert the desired effect on these microorganisms [3]. Even prolonged courses of antimicrobial therapy may not effectively eradicate microorganisms present in biofilm, and hence, infections involving biofilm are prone to relapses after withdrawal of antimicrobial therapy [4]. The protective environment that biofilm offers matures over a course of days to weeks, implying that the duration for which an infection has existed is an important determinant of the outcome of antibiotic treatment [4].

The inherent properties of biofilm have led to the emergence of several principles for the treatment of PJI. First, surgical source control with extensive debridement is an essential part of treatment. Obtaining source control for biofilm infections presenting in an early stage (generally acute infections with an immature biofilm) may be restricted to surgical debridement with implant retention (DAIR) [5]. Achieving source control for chronic biofilm infections (generally assumed to be in existence for more than 3 to 4 weeks) often requires complete removal of the foreign body due to the presence of a mature biofilm. Once the biofilm achieves maturity, persister cells with tolerance towards antibiotics are present, and surgical debridement and systemic antimicrobial therapy alone will not be able to eradicate the infection. Second, select antimicrobials with activity within a biofilm are needed [6]. Third, the duration of antimicrobial therapy is prolonged and often extends to a treatment duration of three months, depending on the surgical strategy [6, 7]. In this paper, we will describe the behavior of bacteria within a biofilm and what characterizes anti-biofilm active antimicrobials based on *in vitro* and clinical data. We will also focus on the current state of knowledge of treatment duration and the role of intravenous (IV) *vs*. oral antibiotic therapy in treating biofilm infections.

## What is a biofilm and how do bacteria behave in it?

Biofilm is a complex network formed between bacteria, the prosthetic material and the host. Aggregates of bacteria (possibly multiple species) form a polymer matrix, which consists of excreted polysaccharides, proteins and DNA. This matrix may also be supplemented by materials from the host, *e*.*g*., in the case of a mounted immune response [2]. Biofilm is characteristic of chronic infections, and it offers a protection against the host immune response and the action of antibiotics [8]. Bacteria embedded in chronic biofilms are in direct contrast with the free-floating, planktonic form of bacteria normally seen in acute infections, although bacteria may once again release themselves from a biofilm in the planktonic state. In PJI, biofilm is importantly associated with the presence of an abiotic surface (the foreign body, the implant), but aggregates and/or biofilms are also seen around biotic surfaces or without a direct relation to a surface [2]. Biofilm has a concentration gradient of nutrients and oxygen relative to the depth: the microorganisms present in the inner core are metabolically inactive and non-dividing [9].

Due to these characteristics, there are several factors that contribute to the antibiotic tolerance of bacteria in biofilm:

- Antibiotics may have difficulty diffusing through the matrix of the biofilm and reaching effective concentrations, especially in the inner core. This, however, is a temporary effect as an equilibrium is eventually reached with antibiotic treatment. Nevertheless, slowly rising antibiotic concentrations may facilitate the build-up of antibiotic tolerance [10].
- Components of the matrix of the biofilm may scavenge antibiotics, rendering them inactive. This has been described with alginate polysaccharides produced by *Pseudomonas aeruginosa* and tobramycin [10].
- The biochemical conditions in the biofilm may result in reduced activity of antibiotics [3]. For example, aminoglycosides do not work adequately in an anaerobic environment.
- Microorganisms inside the biofilm are dormant and do not divide actively [3]. This makes β-lactam antibiotics ineffective, as they require dividing microorganisms to exert their bactericidal effect.
- Microorganisms in the biofilm may show adaptive resistance, *e*.*g*., by upregulating β-lactamase production or efflux pumps. These mechanisms are generally not biofilm-specific, although circumstances may facilitate their emergence, and biofilm-specific adaptive resistance mechanisms have been described [10].
- Lastly, acquired resistance may play a role in biofilm infections as in any other infections. In the case of biofilm infections, the issue is complicated by the fact that the environment may increase the frequency of genetic mutations and facilitate horizontal gene transfer [4].

These mechanisms of antibiotic tolerance have been particularly well studied in *Pseudomonas aeruginosa*, due to its role in biofilms in cystic fibrosis [4]. *P. aeruginosa* has an impressive arsenal of adaptive resistance mechanisms available, but relatively little is known about how well the resistance mechanisms apply to the much more commonly encountered Gram-positive and other Gram-negative microorganisms than *P. aeruginosa* in PJI [11].

## What are biofilm-active antibiotics?

An "anti-biofilm active" antibiotic is classically considered as an antibiotic that penetrates well into the biofilm and demonstrates its capability to eradicate the bacteria embedded within it. In addition, for PJI treatment, it is important that the antibiotic penetrates well into the bone and, for oral antibiotics, has adequate bio-availability [6]. For example, β-lactam antibiotics, especially in their oral formulation, are generally not recommended as the mainstay of treatment. β-lactams have limited oral bio-availability and are generally considered bactericidal. Hence, their activity in chronic biofilms is hampered by bacteria not actively dividing [12].

### Data from *in vitro* studies

Several biofilm models have been developed to assess the property of antibiotic tolerance of microorganisms in biofilms. Concentrations at which antibiotics show efficacy within a biofilm can be quantified by using parameters such as the minimal biofilm inhibitory concentration (MBIC) and minimal biofilm eradication concentration (MBEC) [13].

Molina-Manso et al. quantified MBEC's for several antibiotics used in the treatment of staphylococci on a collection of 32 clinical isolates from PJI [14]. Their study showed that, for compounds such as cloxacillin, vancomycin, clindamycin, co-trimoxazole, ciprofloxacin and daptomycin, median concentrations necessary to eradicate biofilms were generally over 1,024 mg/L, whereas a large proportion of the isolates tested susceptible using conventional MIC testing [15]. Even for the most biofilm-active antibiotic, rifampicin, the median MBEC was 32 and 64 mg/L for *Staphylococcus epidermidis* and *Staphylococcus aureus*, respectively. These concentrations are nowhere near feasible with systemic therapy in *in vivo* situations.

It is important to recognize the shortcomings of such quantifications to mimic the *in vivo* condition. In the commonly used Calgary Biofilm Device (nowadays known as MBEC^®^ assay), biofilm matured during an overnight incubation on an abiotic surface, in a situation with continuous exposure to the medium, but without the complex host anatomy and immune response [8]. In addition, these biofilms were only exposed to antibiotics for a second overnight incubation, whereas in real life, these chronic infections are treated with source control and weeks to months of antibiotic therapy. Assays of biofilm activity have not been adopted in routine laboratory testing due to these discrepancies, combined with the lack of standardization and the fact that enormously high MBECs obtained have no application under *in vivo* conditions [16].

Nevertheless, these in vitro quantifications have made important contributions. For example, they have drawn attention to providing high local concentrations of antibiotics as a part of biofilm treatment regimens, as these may approximate MBEC's in *in vivo* situations [3]. Also, the comparatively lower MBEC’s of rifampicin provide evidence for its crucial role in the treatment of staphylococcal infections. Nevertheless, evidence obtained from in vitro and experimental animal studies should be seen in conjunction with data from human studies. Similar patterns of lower MBEC's, or other quantifications of biofilm activity, may serve as indications for further research for the clinical usefulness of specific (new) compounds or compound combinations [16].

### Data from clinical studies

#### Rifampicin as a biofilm active antibiotic

Over the last decades, rifampicin has emerged as a cornerstone in the treatment of staphylococcal PJIs. Rifampicin penetrates well into tissues, including bone, achieves good intracellular concentrations, and has bactericidal activity in bacteria that are not dividing [17]. It can also be usually prescribed orally due to its excellent oral bioavailability. A microbiological disadvantage of rifampicin is its rapid induction of resistance, which is why (1) rifampicin should always be used in combination with another active anti-staphylococcal antibiotic and (2) it is recommended to start rifampicin after surgical source control when the bacterial inoculum has decreased [18, 19]. Opinions differ on whether the concomitant anti-staphylococcal therapy should have biofilm activity as well, or whether it is merely given to prevent the emergence of resistance with rifampicin being the mainstay of treatment, a notion that would open up a wider array of compounds. The choice of the best co-drug is complicated by the interaction between rifampicin and several antibiotics as a consequence of the cytochrome P450 system induction, but the clinical relevance of these interactions is not always clear. The most optimal companion antibiotic of rifampicin has been analyzed in a multinational non-experimental study on 669 cases of acute early or late staphylococcal PJI, of which 407 received treatment with rifampicin [19]. Unadjusted percentages of treatment failure were 12–14% with levofloxacin, moxifloxacin and clindamycin, 20–21% with β-lactams, linezolid and minocycline, and 38% with co-trimoxazole. These findings strengthen the position of fluoroquinolones as first-line companion antibiotics.

#### The role of rifampicin in staphylococcal PJI

Rifampicin has shown its activity in *in vitro* studies, animal studies and non-experimental clinical studies for staphylococcal PJI [20]. The clinical efficacy of rifampicin in the treatment of orthopedic device-related infections was established by a randomized clinical trial (RCT) published in 1998 on acute staphylococcal infections. This study stopped early after an interim analysis due to the clear superiority of combination therapy with rifampicin compared with ciprofloxacin monotherapy [21]. However, its interpretation is problematic since ciprofloxacin monotherapy would nowadays not be considered an optimal therapy for deep-seated staphylococcal infection, due to the low barrier for resistance emergence. The new generation of fluoroquinolones with more potent Gram-positive activity has largely replaced ciprofloxacin for this indication [22].

A second RCT on rifampicin combination therapy for acute staphylococcal PJI was published in 2020 and questioned the need for rifampicin. This study did not show a benefit compared to vancomycin or cloxacillin monotherapy [23]. The trial has been criticized for (1) not separating out early postoperative infections and late hematogenous infections, (2) differences between the trial registration protocol and the manuscript with regard to the description of the thoroughness of the surgical treatment, (3) the unusual dosage of rifampicin (300 mg *t.i.d*. instead of more commonly 450 mg *b.i.d*.), (4) a lack of monitoring compliant intake of rifampicin, (5) the low statistical power of the study, (6) a lack of detailed description of the failures, and (7) the choice of the companion antibiotic of rifampicin (vancomycin or cloxacillin) [24]. Apart from *in vitro* data and the RCT by Zimmerli *et al.*, many observational studies indicated a clear benefit of rifampicin on clinical outcomes in staphylococcal PJIs [22, 25]. Like many observational studies, analyses are compounded by confounding bias (rifampicin may be reserved for more complicated cases), competing risks (impeding failure may prevent the initiation of rifampicin treatment), and appropriate categorization of rifampicin treatment based on starting point and duration. Despite these limitations, rifampicin is still considered the first-line antibiotic treatment for staphylococcal infections with the currently available evidence.

#### The role of rifampicin for other Gram-positive microorganisms

Rifampicin has also been considered as a treatment adjunct in streptococcal and *Cutibacterium spp.* PJIs. Commonly, β-lactams and clindamycin are used for these infections, but they demonstrated limited *in vitro* activity against biofilm-embedded bacteria, while rifampicin showed better *in vitro* activity [26–29]. For both streptococcal and *Cutibacterium spp*. PJIs, the only available clinical studies are observational.

Lora-Tamayo *et al.* performed a multinational study of 462 cases of streptococcal PJI treated with DAIR, of which 52% were hematogenous infections and the remainder were post-surgical ones [30]. The authors showed that the cumulative duration of rifampicin use in the first 30 days post-surgery was associated with lower failure rates, and suggested that either prescription of a β-lactam or rifampicin was necessary for an optimal outcome.

In another multinational study, Kusejko *et al.* analyzed 187 cases of *Cutibacterium spp*. PJI, of which 85% was *C. acnes*. The infections generally involved the shoulder or hip, and 95% presented with a chronic infection at least a month after implantation [31]. In each of the three main subgroups of surgical approaches (one- and two-stage revision, and DAIR), the addition of rifampicin to the antibiotic regimen was associated with better clinical outcomes in the unadjusted analyses. In the adjusted analysis of treatment failure in the complete cohort, rifampicin use was protective with a hazard ratio (HR) of 0.5, but the 95% confidence interval crossed 1. This effect size was similar in magnitude to the detrimental effect of the use of DAIR on the outcome of these chronic infections (HR 2, statistically significant), indicating that the surgical approach in Cutibacterium infections is more important than the selected antimicrobial therapy. All in all, there may be clinical benefit to combination treatment with rifampicin in selected cases of streptococcal and Cutibacterium PJI, but it is not routinely recommended.

#### The role of fluoroquinolones in Gram-negative microorganisms

In Gram-negative PJI, the use of ciprofloxacin is generally considered pivotal for eradication of biofilm, although there is limited evidence from *in vitro* and animal studies. *In vitro* data in which fluoroquinolones showed the highest biofilm eradication rate when compared to other antibiotics was mostly demonstrated in *Pseudomonas aeruginosa* biofilms [32, 33], but has also been observed for *Escherichia coli* [34] and *Stenotrophomonas maltophilia* [35].

The largest observational study has been published by Rodriguez-Pardo *et al.* and included 174 cases treated with DAIR, of which 78% were caused by Enterobacterales, and 20% by *Pseudomonas aeruginosa* [36]. Treatment with ciprofloxacin had a clinical success rate of 79%, whereas treatment with other antibiotics in the cases of both ciprofloxacin susceptibility or ciprofloxacin non-susceptibility resulted in success rates around 40% (*P* < 0.001). It is important to note that only ten patients in this study were treated with co-trimoxazole, and therefore, the comparative effectiveness of ciprofloxacin and co-trimoxazole as oral alternatives remains to be established. A smaller observational study (*n* = 47) confirmed the inferiority of other oral antibiotics to ciprofloxacin [37]. The only direct comparison that has been made between an oral fluoroquinolone and an alternative regimen was with intravenous β-lactams [38]. In this French study, patients who could not be treated with fluoroquinolone remained on IV β-lactams during the whole treatment period with or without another co-antibiotic. The clinical outcome between both groups was similar.

## Duration of antibiotic therapy

Traditionally, treatment duration for PJI has been at least 3 months, which is substantially more than the 4 to 6 weeks that is generally advised in the case of the absence of orthopedic hardware [39]. This is motivated primarily by the difficulty to eradicate bacteria embedded in biofilm.

Bernard *et al.* performed the multicenter DATIPO RCT in France, in which 410 patients with a PJI of the hip or knee were randomized to either 6 or 12 weeks of antibiotic therapy after surgical source control [7]. The primary endpoint;??? persistence or relapse of infection within 2 years after antibiotic therapy withdrawal occurred in 18.1% of those treated for 6 weeks *vs*. 9.4% of those treated for 12 weeks, with the confidence interval of the risk difference excluding the non-inferiority of 6 weeks of treatment. In subgroup analyses, the inferiority of a 6-week antibiotic regimen was particularly evident in patients treated with DAIR, with an absolute risk difference of 16.2%. The primary endpoint was not significantly different for patients who were treated with a one- or two-stage revision. This finding supports the notion that the presence of infected orthopedic hardware is an important factor in the prognosis of PJI treatment. The RCT performed by Benkabouche *et al.* even demonstrated that recurrences did not differ between 4 and 6 weeks of antibiotic treatment after the removal of orthopedic hardware [40]. However, it must be noted that one-stage revision surgeries were not included in this trial, and, therefore, the treatment duration after one-stage exchange arthroplasty remains a matter of debate.

A Spanish multicenter RCT by Lora-Tamayo compared an 8-week antibiotic regimen with 3- or 6-month regimens for staphylococcal PJI of the hip or knee treated with DAIR [41]. In the intention-to-treat analysis, the short regimen was non-inferior regarding clinical cure. It should be pointed out that, in this trial, patients were only included if they could be treated with a fluoroquinolone combined with rifampicin, and in addition, cases with an initially poor prognosis with a high odds of developing early failure were excluded.

Although uncertainties remain based on the above-mentioned studies, all in all, there is potential for shortening antibiotic treatment duration to 6 weeks, in particular, for selected groups of patients with an acute PJI and a low risk of failure after DAIR, and for chronic infections treated with revision surgery. Results are awaited of the SOLARIO trial. This trial evaluates whether 7 or fewer days of systemic antibiotics is non-inferior to a long course of antibiotic treatment if local antibiotics have been applied. Although the study is designed for orthopedic infections in general, making it more difficult to evaluate subgroups with sufficient statistical power, cases with a PJI are included as well [42].

## When is it safe to switch to oral therapy?

A final recurring question with regard to the treatment of PJI is at what time point a switch from intravenous to oral treatment is justified. Traditionally, osteomyelitis and PJI were considered an entity that should be treated with intravenous antibiotics only [43]. However, as previously discussed, many compounds considered critical in the treatment of PJI (rifampicin, fluoroquinolones) have excellent bio-availability and do not rely on high plasma concentrations for bone penetration.

Early oral therapy in the cases of osteomyelitis and PJI has received momentum in recent years through the OVIVA trial. In this British multicenter RCT, 1054 patients with bone and/or joint infection were randomized to either a switch to oral therapy within 7 days after surgery, or a full 6-week course of intravenous antibiotics and, if needed, followed by additional oral therapy [44]. In 61% of randomized patients, the infection was related to a foreign body, including prosthetic joints, and was surgically managed with DAIR, definite removal of the implant, or one- or two-stage exchange. Early oral treatment, achieved in approximately 90% of patients randomized to this arm, was non-inferior with regard to treatment failure as compared to a long course of intravenous antibiotics. This trial supports the early switch to oral antibiotics, even in complex orthopedic implant-related infections, provided that adequate surgical debridement has been achieved and an oral antibiotic with antibiofilm properties and sufficient bioavailability is available. Guidelines on the type of targeted antibiotics and corresponding dosage have been previously described [45, 46].

## Conclusions

- Antimicrobial tolerance, developing during maturation of the biofilm, makes PJIs difficult to treat and necessitates adequate source control, antimicrobial therapy with "anit-biofilm active" antibiotics and prolonged courses of antibiotic therapy.
- Applying biofilm models for susceptibility testing in routine laboratory testing is currently not clinically useful. Ideally, though, in the future, individual characteristics of the biofilm related to the bacterial strain and the manner of the biofilm being embedded into the host would shape antibiotic choices.
- Biofilm-active therapy in the case of *Staphylococci* consists of rifampicin, supplemented with a second antibiotic to prevent resistance formation. Most experience exists with using fluoroquinolones with proper Gram-positive action as companion drugs (*i*.*e*., levofloxacin, moxifloxacin), but other antibiotics with good bio-availability and bone penetration may just be as adequate.
- Rifampicin for *Streptococci* and *Cutibacterium spp*. showed good activity in *in vitro* biofilms. Observational studies suggested that it might increase clinical cure, but the surgical strategy might be a more important predictor for clinical outcomes. Routine use is not recommended, but it might be considered in certain subgroups of patients.
- Fluoroquinolones show good *in vitro* activity against biofilms produced by certain Gram-negative organisms. In addition, observational studies reported good outcomes using this compound, and is, therefore, still the first-line antibiotic treatment for PJIs caused by Gram-negatives.
- Antibiotic treatment duration should be 3 months for acute PJIs treated with DAIR but may be shortened for the cases with a low risk of failure. In the case of revision surgery, 6 weeks of antibiotic treatment probably suffice.
- IV antibiotics can be switched early to an oral treatment regimen, provided that an antibiotic with anti-biofilm properties and adequate bioavailability is available.

## Data Availability

Not applicable.
